# Evaluating patient perspectives on participating in scientific research and clinical trials for the treatment of spinal cord injury

**DOI:** 10.1038/s41598-021-83211-2

**Published:** 2021-02-23

**Authors:** Soukaina Bahsoun, Jan-Herman Kuiper, Charlotte H. Hulme, Angus J. Armstrong Twigg, Wagih El Masri, Clive Glass, Bakul Soni, Naveen Kumar, Joy Roy Chowdhury, Aheed Osman, Karina T. Wright

**Affiliations:** 1 School of Pharmacy and Bioengineering, Keele University, RJAH Orthopaedic Hospital, Stoke-on-Trent, UK; 2 Robert Jones and Agnes Hunt Orthopaedic Hospital Foundation Trust, Oswestry, Shropshire SY10 7AG UK; 3 Southport and Formby General Hospital, Southport, Merseyside PR8 6PN UK

**Keywords:** Stem cells, Medical research, Neurology

## Abstract

A questionnaire was developed to evaluate patients’ perspective on research aimed at improving functions and overcoming complications associated with spinal cord injury (SCI). The first three sections were based on published and validated assessment tools. The final section was developed to assess participant perspectives on research for SCI. One thousand patients were approached, of which 159 participated. Fifty-eight percent of participants were satisfied with their ‘life as a whole’. Two factors could be generated that reflected the variance in the data regarding participants’ life with a SCI: “*Psychosocial and physical wellbeing*” and “*Independent living*”. The majority of participants stated they would be involved in research (86%) or clinical trials (77%). However, the likelihood of participation dropped when potential risks of the research/trials were explained. Which participants would be willing to participate in research could not be predicted based on the severity of their injury, their psychosocial and physical wellbeing or their independent living. Despite participant establishment of a life with SCI, our data indicates that individuals strive for improvements in function. Participant willingness to be included in research studies is noteworthy and scientists and clinicians are encouraged to involve more patients in all aspects of their research.

## Introduction

The development of new treatments and therapies for spinal cord injury (SCI) is an active area of health research. The ongoing research into SCI has a definitive aim to improve the quality of life for individuals with SCIs. However, what in research is important to individuals with SCIs is rarely established and can as a result be overlooked.

A study of 14 patients based around an evidence-based questionnaire found that individuals with SCI may feel more capable and autonomous if they access information related to research^1^. Furthermore, the study concluded that all participants were interested in learning about research and clinical trials^1^. Others have ascertained that most research questions are formulated within academic institutions where SCI patients themselves have no direct involvement; consequently research foci may shift away from fulfilling the needs and the preferences of SCI patients^2^. Although a harmony exists between the patients’ and researchers’ interests, the study suggests that the SCI research community should be more inclusive of patients in order to not only meet their demands but also to enlarge the scope of applications when novel knowledge is obtained through research^2^.

A subsequent survey of a SCI population required participants to rank seven bodily functions that are important for the improvement of their quality of life^3^. The authors suggest that the results of this survey could help scientists and clinicians in the field to target an area of research that has a direct effect on SCI patients’ quality of life, unlike much current research that is directed towards “curing” SCI while ignoring the need for independence, psychological, social and economic life of individuals with SCI^3^. Another study suggested that the competencies of individuals with SCIs must be emphasised, and their experiences must be appreciated by researchers and used to formulate research questions with the greatest potential for patient benefit^4^. Moreover, this study stresses the need for research outcomes that influence patients’ lives (directly or indirectly) within a short timeframe^4^.

Together these publications indicate that the opinions, values, expectations and fears of individuals with an SCI can be a valuable resource for scientists and clinicians in this field of research but are frequently overlooked. Our study aims to ensure that patient opinion is considered in future scientific study and clinical trial development. Our specific objectives are to assess (1) the willingness of SCI patients to participate in various types of SCI research, and (2) the correlation between their willingness to participate with injury severity, perceived quality of life or psychological wellbeing.

## Results

### Participants

Questionnaires were returned by 159 participants (15.9%). The majority of participants were male (114 males cf. 43 females (2 undisclosed)) and their mean age was 45 years ± 18SD (range 20–80). Sixty participants were classed as American Spinal Injury Association (ASIA) Impairment Scale (AIS) grade A, 18 AIS grade B, 28 AIS grade C and 38 AIS grade D on admission with data not being held for the admission scores of 15 patients. Twenty four patients showed an improvement in their AIS grade at the time of discharge cf. their score on admission, the vast majority (58%) of which improved from AIS grade C to grade D. The mean time of injury to questionnaire completion was 10 ± 9SD years, with 88.7% having lived with their SCI for two or more years.

Keeping within the ethical constraints of this study, some data was available for inclusion in this report on all of the invited participants who had been patients at the MCSI, in terms of demographic information, time of injury and neurological grade as assessed using the Frankel classification. This information was used only to demonstrate that the patients who returned questionnaires were representative of the invited cohort. In the invited cohort, 69% of the participants were males with similar proportions of the participants being male (64%). There was a wide age range of participants (at the time of response and time of injury), which again represented the invited population (Table 1). Further, individuals participated from all different injury severities (Table 1). Individuals participated in the study irrespective of whether they demonstrated an improvement in severity, as deemed by one Frankel grade change, from time of injury to time of discharge, again representing the overall invited cohort (invited improvers, 9%; participated improvers, 16%).

**Table 1:** Demographic and injury information of individuals who were invited and who participated in the study from the Midlands Centre for spinal injury.

	Invited % (N) N = 104	Participated % (N) N = 499
Male	64 (67)	69 (346)
**Age at time of questionnaire completion (years)**		
> 20–< 30	3 (3)	6 (29)
> 30–< 40	15 (16)	11 (54)
> 40–< 50	18 (19)	18 (89)
> 50–< 60	27 (28)	27 (135)
> 60–< 70	18 (19)	19 (93)
> 70–< 80	13 (14)	12 (62)
> 80–< 90	5 (5)	6 (29)
Unknown	1 (1)	1 (0)
**Age at time of SCI (years)**		
< 20	8 (8)	10 (51)
> 20–< 30	25 (26)	24 (122)
> 30–< 40	13 (14)	14 (72)
> 40–< 50	15 (16)	15 (77)
> 50–< 60	13 (14)	14 (72)
> 60–< 70	11 (11)	8 (39)
> 70–< 80	11 (11)	7 (34)
> 80–< 90	0 (0)	2 (8)
Unknown	4 (4)	5 (24)
**Years since SCI (years)**		
< 2	3 (3)	4 (20)
> 2–< 5	20 (21)	19 (97)
> 5–< 10	23 (24)	19 (93)
> 10–< 20	31 (32)	26 (131)
> 20–< 30	8 (8)	15 (73)
> 30–< 40	9 (9)	11 (55)
> 40	1 (1)	1 (4)
Unknown	6 (6)	5 (26)
**Injury severity on admission (Frankel)**		
A	50 (52)	49 (243)
B	18 (19)	14 (69)
C	24 (25)	28 (140)
D	4 (4)	4 (21)
Other/unknown	4 (4)	5 (26)

### Participants’ opinions on scientific research, experimental treatments and clinical trials for SCI

**Figure 1:**
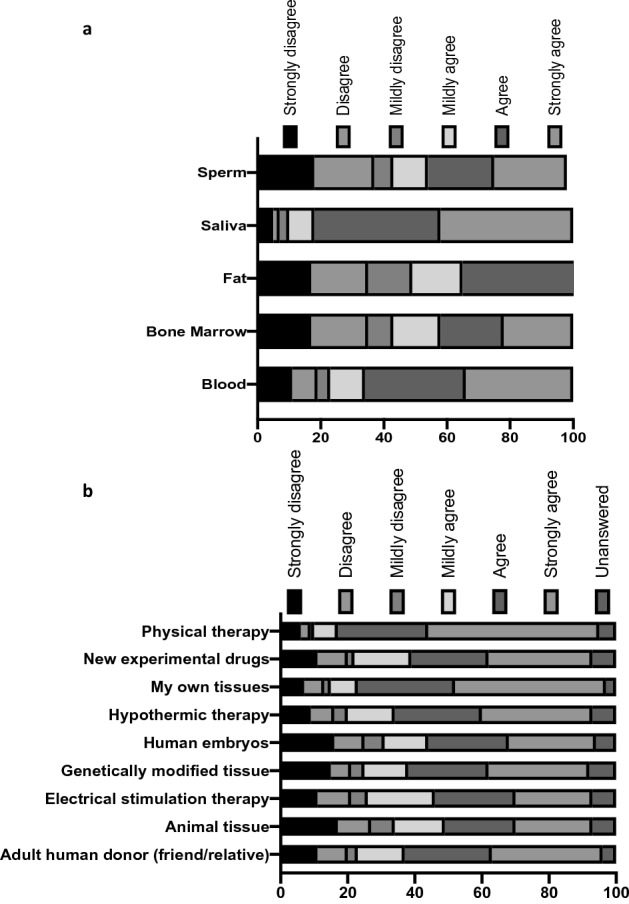
(**a**) Bar graph displaying participant responses to the statement: “I would like to be more involved in scientific research, including donating the following for research purposes:” (**b**) Bar graph displaying participant responses to the statement: “I would consider taking part in an experimental treatment or clinical trial that involved the use of”.

Participants were receptive to research with 81% stating they would be happy for their medical records to be accessed for scientific research. Figure 1a illustrates that the majority of participants would at least “Mildly Agree” to donating tissue to research, with the lowest participation coming from the response to donation of bone marrow tissue (54.9% mildly agree or more). There was an overarching willingness to participate in all of the experimental treatments and clinical trials suggested including the use of human, animal and genetically modified tissues and cells (Fig. 1b).

**Figure 2:**
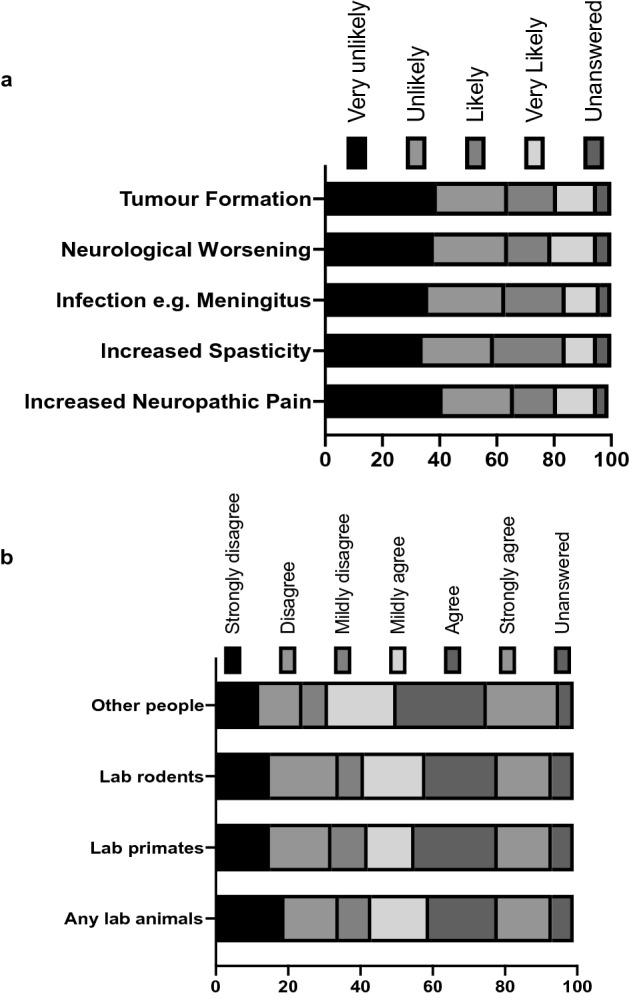
(**a**) Bar graph showing participant response to the question: “If the following risk of a treatment was explained to you by a clinician before volunteering for an experimental treatment or clinical trial, how likely would you be to take part?”. (**b**) Bar graph showing participant response to the statement: “I would be prepared to take part in an experimental treatment of clinical trial which had NOT first been tested on”.

Only in response to having associated risks (tumour formation, infection, neurological worsening of neurology, increased spasticity and increased neuropathic pain) being explained alongside the prospective trial/treatment did the questionnaire participants’ responses demonstrate a pattern skewed towards a unlikely participation (Fig. 2a). Perhaps surprisingly, there was a large number of participants who were willing to be part of experimental treatment or a clinical trial NOT first tested on: lab animals in general, primates, rodents, or other humans (Fig. 2b).

### Participants’ opinions on living with a SCI

**Figure 3:**
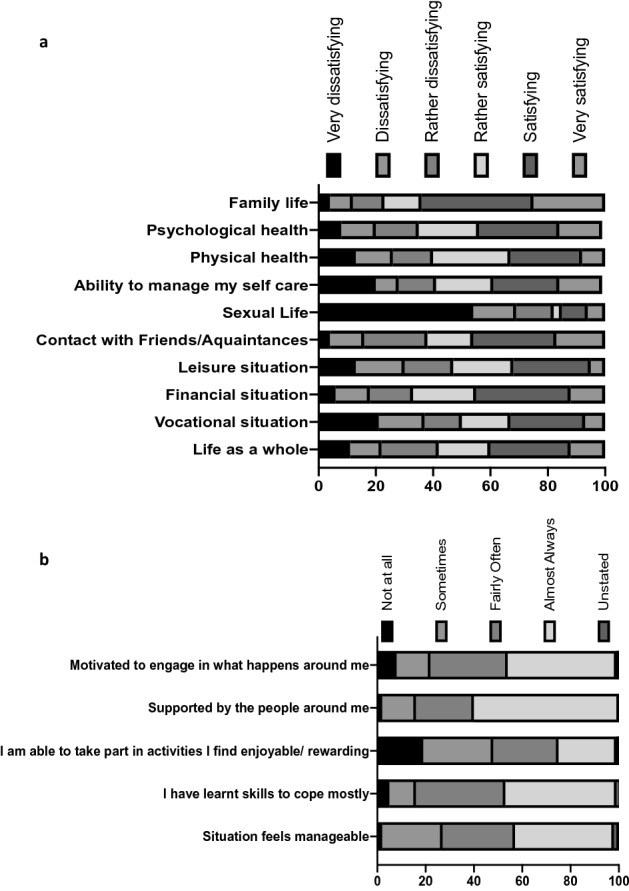
Participants’ responses on a Likert scale to questions about how they feel about their life in general. (**a**) Proportion of each response of the total number of responses about satisfaction with different aspects of life. (**b**) Proportion of different responses about how participants feel about their SCI.

The data from the three Craig Handicap Assessment & Reporting Technique (CHART) summary scores were not normally distributed, but the other six summary scores were. The CHART summary scores for occupation and mobility indicated that at least 75% of participants considered themselves having a disability in these areas by scoring below 100, and scoring particularly low on the occupation score (Table 2). The Life Satisfaction checklist (LISAT)-11 scores indicated that over half the participants (58%) were satisfied with their life as a whole, with sexual life and leisure situation being the aspects most people were dissatisfied with (Fig. 3a). The four LISAT-11 summary scores all had a mean value slightly above the 50% point of their full range (Table 3). The majority of participants believed that their situation was manageable (Fig. 3b and Table 4). Only 25% of the participants scored 12 or less, leaving 75% scoring in the upper half of the scale.

**Table 2:** Percentile range of the three summary scores based on CHART.

Percentile range	25th	50th	75th	100th
Occupation score	10.6	42.5	95	100
Physical independence score	4	72	100	100
Mobility score	58	79	99	100

**Table 3:** Mean and SD f scores based on LISAT-11.

Score	Mean (SD)
Provision	5.8 (2.0)
Spare time	6.3 (2.1)
Closeness	8.5 (3.0)
Health	8.0 (2.8)

**Table 4:** Mean, standard deviation and quartiles of the Manageability score based on PMnac.

Score	Mean (SD)	Range	25th	50th	75th
Manageability	15.0 (3.7)	4–20	12	15	18

### Determining whether participant demographics/injury and their opinions on living with SCI relates to their willingness to participate in research

Before investigating the correlation of participants’ characteristics and their opinions on living with SCI with their willingness to participate in research, we performed two separate factor analyses to reduce the number of items in the questionnaire to a smaller number of independent factors. One factor analysis was performed using the 39 ordinal components in section 4 of the questionnaire. A preliminary analysis using the eigenvalue and factor loading criterion resulted in a seven-factor pattern, but the seven factors were not meaningfully interpretable. A parallel analysis was therefore performed, which suggested retaining five components. The updated factor analysis based on five components contained six items (consent to access records, donating saliva, harvesting blood, lumbar puncture, back surgery and placebo) that did not belong to any of the factors and we therefore removed them. The remaining five-factor solution was interpretable and explained a large percentage (76.2%) of the variance in the data (Suppl. Table 1).

We interpreted the five factors as representing research activities with increasing perceived risk levels or reflecting increasing invasiveness, ranging from level 1 (completely safe participation) to level 5 (highest risk). Participation level 1 was termed *‘opinion-based research involvement’* and included group meetings, presentations, postal communications and questionnaires. Participation level 2 was *‘tissue donor involvement’* including donating blood, fat, bone marrow, sperm and harvesting fat, bone marrow and tissue from olfactory bulb. Participation level 3 was *‘clinical trial involvement (without risk explained)’*, which included the administration of cells from various sources, new experimental drugs, electrical stimulation, hypothermic and physical therapy. Participation level 4 was *‘clinical trial involvement (without prior testing)’*, which represented taking part in clinical trials not tested before on other people, lab primates, lab rodents or lab animals. Participation level 5 was *‘clinical trial involvement (with risk explained)*’ which represented taking part in clinical trials even when associated risks i.e. tumour formation, infection, neurological worsening, increased spasticity and increased neuropathic pain were explained. Participation level 3 represented by far the largest amount of variance (46%) of the data, whereas the other four levels explained less than 10% each (Suppl. Table 1). Using the factor loadings, we determined the theoretical range for each of the five factors and their values for each participant. The median scores were well above the midway point for the lowest three levels, suggesting that the majority of participants were willing to participate in research at these levels. However, the median score was only slightly above the midway point at the fourth level and clearly below the midway point at the highest level, indicating that participants were more hesitant to participate at the highest perceived risk levels.

Spearman analysis was used to assess internal correlations of the nine summary scores on life with SCI, which all correlated significantly with each other (range of r 0.16–0.68) A factor analysis was performed, resulting in a two-factor pattern that explained 64.0% of the total variance in the data (Suppl. Table 2). The first factor consisted of spare time, life as a whole, manageability, provision, health and closeness and was entitled “*Psychosocial and physical wellbeing*”. The second factor encompassed physical independence, occupation and mobility and was labelled “*Independent living*”.

Scores for each participant were calculated for the two factors representing life with SCI, based on the loading of items in each factor, and used to examine how willingness to participate related to the participants’ demography, level of injury and opinion on life with SCI (Table 5). No correlations were observed between the factors relating to participant general wellbeing and independent living and their willingness to participate. There was a negative correlation between sensory ASIA score and participation level 3 (clinical trial involvement (without risk explained)) (p < 0.01; Table 5). The participant demographics which had the greatest impact on the participants willingness to participate in research was age, which correlated (negatively) with participation level 1 (p < 0.05; Table 5), participation level 2 (p < 0.01; Table 5), participation level 3 (p < 0.05; Table 5) and (positively) with participation level 5 (p < 0.05; Table 5). Age of the participants, as well as being female, also negatively correlated with psychosocial and physical wellbeing and independent living factors (p < 0.001; Table 5).

**Table 5:** Correlation of injury status (ASIA motor and sensory scores), psychosocial and physical wellbeing and independent living with level of willingness to participate in research.

	AM	AS	Gender—female	Age	PP	IL	L1	L2	L3	L4	L5
AM	1										
AS	0.68***	1									
Gender	0.068	0.186*	1								
Age	0.068	0.304***	0.028	1							
PP	0.135	0.112	− 0.733***	− 0.567***	1						
IL	− 0.03	0.57	− 0.583***	− 0.476***	0.411***	1					
L1	− 0.034	− 0.017	0.065	− 0.166*	0.075	0.148	1				
L2	− 0.065	− 0.15	− 0.229**	-2.960***	0.021	0.128	0.457***	1			
L3	− 0.107	− 0.216**	− 0.152	− 2.036*	0.004	0.067	0.398***	0.675***	1		
L4	0.117	0.038	− 0.087	0.156	0.088	0.07	0.342***	0.414***	0.569***	1	
L5	− 0.032	− 0.091	0.152	0.168*	− 0.136	− 0.054	0.256***	0.517***	0.505***	0.465***	1

AM, ASIA motor score; AS, ASIA sensory score; IL, independent living score; PP, psychosocial and physical wellbeing score; L1, participation level 1—*‘opinion-based research involvement’*; L2, participation level 2— *‘tissue donor involvement’*; L3, participation level 3— *‘clinical trial involvement (without risk explained)’*; L4, participation level 4— ‘*clinical trial involvement (without prior testing)’*; and L5, participation level 5—*‘clinical trial involvement (with risk explained)’*. Significance levels: *** < 0.001; ** < 0.01; * < 0.05.

Within each group of factors (willingness to participate and opinion on life with SCI) significant correlations were also found. The five levels of research participation all correlated significantly with each other, as did the psychosocial and physical wellbeing and independent living and factors (p < 0.001; Table 5).

## Discussion

The importance of patient participation in research has been acknowledged by research institutions and policy makers for some time^5^. Our questionnaire was specifically designed to try to address a gap that we identified through our own translational SCI research programme. Our results demonstrate a strong willingness of persons with SCI to participate in research, further underlining the importance of involving SCI patients’ directly. The overall response rate of 15.9% was disappointingly low, however our analysis of the invited vs. participated population indicates that our cohort was representative of the whole. The reason for this low return rate we feel is due to the constraints imposed on recruitment into the study by our ethical review board, who decided that we were only permitted to approach a potential participant once by post.

Participants responded that they would be willing to participate in all aspects of research. However, a shift towards lower numbers was noted in the participants willingness to participate in trials of treatments that had not been tested before, and a marked shift if the trials involved significant potential health risks. This decrease in willingness to participate in research when there was a perceived risk is not surprising. Nevertheless, the majority of participants would still agree to take part in research not first tested on animals or other humans. This could be related to a general critical position in relation to animal testing, but we did not investigate the participants’ general stance on animal experimentation. We also observed that participants were “very unlikely” to be involved in treatments which carried an increased risk of worsening aspects of their morbidity. A 2018 study ^6^ displayed a significant correlation between increased spasticity and decreased quality of life scores, which supports our finding that when the possibility of spasticity is explained, the majority of participants felt this potential risk would not outweigh the potential benefits of taking part in a trial.

Most participants in our study were satisfied or very satisfied with their life as a whole and various aspects of their life, but not with their sexual life, where the vast majority (both genders) were not satisfied. These findings are almost exactly the same as those seen in earlier comparable studies^7,8^. The degree of disability reported by our participants also matches data reported earlier, including the low scores observed in relation to occupation and physical independence^7^. In 2006, people with SCI in the UK reported markedly higher degrees of disability in the domain of physical independence than those in Germany, Australia and Switzerland^7^. Our results therefore suggest that physical independence of people with SCI in the UK has not improved in the intervening years. Finally, our participants reported the same high level of perceived manageability of their situation as found in an earlier study with a similar population^7^.

Overall, 89% of participants in our study had been living with their injury for more than 2 years and our data suggests there is a good level of life satisfaction and a high level of manageability in our participant cohort. However, participants felt clearly disadvantaged when it came to physical independence and occupation. This separation between general (psychosocial and physical) wellbeing and independent living was mirrored in our factor analysis, which suggests these two aspects of life with SCI are largely independent. Nevertheless, we found that both correlated negatively with age and being female. The negative influence of age corresponds to earlier work, which also found that SCI patients reported lower levels of life satisfaction and physical health, and physical independence and occupation function, with increasing age^9^. Likewise, earlier work also found that female SCI patients reported lower living circumstances, mental health and satisfaction with health^10^.

Even though most participants appeared to be satisfied with their life and ability to manage, their readiness to be involved in research was strongly affirmative. We found no evidence for a correlation between independent living or psychosocial and physical wellbeing scores and willingness to participate at any of the perceived risk levels. We also found no evidence for a correlation between willingness to participate and injury status as measured by the ASIA scores, other than a weak negative correlation between sensory score and willingness at level 3. Willingness to participate at level 3 represented almost half the total variation in our data, which probably explains why it was the only level demonstrating some correlation. The negative correlation suggests that people with better sensory scores are less willing to participate. However, the correlation was weak. The five levels of participation that we distinguished provide clues regarding how much an individual is willing to participate in research (with or without perceived risk). All levels of participation correlated positively and strongly with each other, which would suggest the existence of an overarching second-order factor “*willingness to participate in research*”.

Our study aims to assist in bridging the gap for scientists and clinicians from the bedside-to-bench by auditing the opinion of those patients most likely to benefit from many of the proposed interventions currently under development and being translated into the clinical setting. We conclude that individuals with SCI are interested in new research provided they are given an informed choice and are willing to participate provided the health risks are not too high and that researchers must involve patients more actively, providing contact between the research teams and the patients themselves, making research findings more accessible. The assessment strategies developed and described in this study will provide valuable tools for assessing the opinion of the SCI patient community, which should be evaluated in a larger patient cohort in future studies.

## Materials and methods

### Study design

We developed a four-section questionnaire (appendix A) in order to evaluate the opinions of SCI patients from two UK spinal injury units: The Midland Centre for Spinal Injuries (MCSI) [part 1] and the North West Regional Spinal Injuries Centre (NWRSIC) [part 2]. The questions in sections 1, 2 and 3 of the questionnaire were based on published and validated assessment tools, namely the CHART short form^11^, the LISAT-11^12^ and the Perceived Manageability Scale (PMnac)^13^. All questions in section 4 were developed by the authors to assess the participants’ opinions on research and novel treatments for SCI. Ethical consent for the study was given by the Liverpool Central NRES Committee North West (13/NW/0486). All research was performed in accordance with relevant guidelines/regulations, and informed consent was obtained from all participants. The postal questionnaire was sent to 1000 individuals who had been admitted to the MCSI or NWRSIC with a SCI between 1975 and 2014 and who were chronically injured (more than 12 months past the date of their SCI). Questionnaires and consent forms were returned between 2014 and 2016.

### Measures

From sections 1, 2 and 3, nine summary scores were calculated for each participant. Question 5 in section 1 of the questionnaire (based on CHART-SF^11^) was used to calculate the “Occupation” score. Questions 1 and 2 in section 2 of the questionnaire (also based on CHART-SF^11^) were used to calculate the “Physical Independence” and “Mobility” scores. Each resulted in a score ranging from 0 (worst) to 100 (best), with scores over 100 capped at 100 and implying “no disability”^11^. Question 1 in section 3 (based on LISAT-11^12^) had 11 items and was developed as a checklist of satisfaction with life as a whole and with ten separate domains of life, such as health, leisure and sexual life^12^. The score on each item ranged from 1 (worst) to 6 (best), and the scores on the ten separate domains were summarised using four factors, namely Provision (range 1.6–9.5), Spare time (range 1.6–9.8), Closeness (range 2.4–14.3) and Health (2.1–13.0). The second question in section 3 (based on PMnac^13^) was used to calculate the “Manageability” score, ranging from 5 (worst) to 20 (best). No summary scores were calculated from section 4. Instead, descriptive statistics of each answer were presented to assess SCI patients’ opinions on and willingness to participate in various forms of SCI research. Ordinal answers were used to analyse the correlation between the above characteristics of SCI patients and opinion on or willingness to participate in research.

ISNCSCI is the International Standards for Neurological Classification of Spinal Cord injury. Within the ISNCSCI there are neurological scores and grades, including the ASIA motor score and ASIA sensory score^14,15^. To assess the degree of injury, ASIA scores were obtained for every participant as close as possible to the time of questionnaire completion. The motor and sensory numeric scores were used for these analyses.

### Missing value imputation

To impute missing data where questions were not completed, particularly in the LISAT-11 related sections, a hot deck imputation macro^16^ was used in SPSS vs 22 (IBM UK Ltd, Portsmouth, UK). Based on correlations among the LISAT-11 items, appropriate decks were chosen. Imputation of missing values was conducted in order of the items with the least missing values to the items with the most missing values. When possible three decks were used, otherwise two decks were used.

### Statistical analyses

The results of the survey were summarised using descriptive statistics. Normally distributed continuous data was summarised as mean (SD), ordinal data as median (IQR) and range, and nominal data as frequency and percentage. A correlation analysis was used to analyse whether or not the patients’ views on and willingness to participate in scientific research, experimental treatment and trials for SCI correlated with their age, gender, SCI status (as measured by sensory and motor ASIA scores) and the patients’ assessment on how SCI affected them. Before the correlation analysis, we performed two separate factor analyses (one of the nine summary scores from sections 1–3 and one of the 39 ordinal items from section 4 of the questionnaire) to obtain a more parsimonious set of independent factors^17^. All factors with an eigenvalue greater than 1 and an item loading greater than 0.3 were retained, and a Direct Oblimin rotation was used. If the retained factors could not be readily interpreted, we used Parallel Analysis to determine the number of factors to be retained^18^. All correlation analyses, including the two factor analyses, used Pearson’s correlation coefficient if data was normally distributed as judged from QQ-plots, otherwise Spearman’s rank correlation was used. Statistical analyses were performed using SPSS 22 (IBM UK Ltd, Portsmouth), R v 3.6.0 (The R Foundation for Statistical Computing, Vienna, Austria) and Monte Carlo PCA for Parallel Analysis^19^. A 2-sided p-value below 0.05 was assumed to denote statistical significance.

## Supplementary Information

Supplementary Information.
