# Supplementary material for: Evaluating patient perspectives on participating in scientific research and clinical trials for the treatment of spinal cord injury
Source: Sci Rep. 2021 Feb 23;11:4361. doi: 10.1038/s41598-021-83211-2 (PMC7902830; doi:10.1038/s41598-021-83211-2)
Supplement: Supplementary file 1 — Supplementary Information. [file 41598_2021_83211_MOESM1_ESM.doc]

**Evaluating Patient Perspectives on Participating in Scientific Research and Clinical Trials for the Treatment of Spinal Cord Injury**

Soukaina Bahsoun^1,2*^, Jan-Herman Kuiper^1,2*^, Charlotte H Hulme^1,2^, Angus J Armstrong Twigg^2^, Wagih El Masri^2^, Clive Glass^3^, Bakul Soni^3^, Naveen Kumar^2^, Joy Roy Chowdhury^2^, Aheed Osman^2^, Karina T Wright^1,2^ ,

1. School of Pharmacy and Bioengineering, Keele University, Stoke-on-Trent

2. Robert Jones and Agnes Hunt Orthopaedic Hospital Foundation Trust, Oswestry, Shropshire, SY10 7AG

3. Southport and Formby General Hospital, Southport, Merseyside, PR8 6PN

*Indicates equal author contribution.

Corresponding author: Dr Karina T Wright, School of PhaB, Keele University, RJAH Orthopaedic Hospital. E-mail: karina.wright1@nhs.net

**Supplementary Table 1: Five-component factor analysis of the willingness to participate in research questionnaire items, with the number of factors decided by parallel analysis and using** Direct Oblimin rotation.

| **Component** | **Eigenvalue** | **% Variance explained** | **Theoretical range** | **Median (IQR)** |  |
| --- | --- | --- | --- | --- | --- |
| ***Participate level 1*** | 1.5 | 4.6 | 2.9-17.9 | 14.1 (11.1-15.7) |  |
| ***Participate level 2*** | 2.7 | 8.3 | 5.2-31.3 | 21.9 (14.3-27.8) |  |
| ***Participate level 3*** | 15.3 | 46.5 | 9.6-57.8 | 47.3 (35.7-55.2) |  |
| ***Participate level 4*** | 2.4 | 7.4 | 3.6-21.5 | 14.4 (7.2-18.0) |  |
| ***Participate level 5*** | 3.1 | 9.4 | 4.5-18.2 | 9.1 (4.5-12.3) |  |

**Supplementary Table 2: Factor analysis of the nine summary scores reflecting life with spinal cord injury. N= 163.** Eigenvalue greater than 1, item loading greater than 0.4 and Direct Oblimin rotation.

| **Component** | **Rotated Loading** | **Eigen value** | **% Variance explained** |
| --- | --- | --- | --- |
| **Psychosocial and physical wellbeing**  Spare time  Life as a whole  Manageability  Provision  Health  Closeness | 0.9  0.8  0.8  0.8  0.7  0.6 | 4.5 | 49.7 |
| **Independent living**  Physical independence  Occupation  Mobility | 0.9  0.8  0.6 | 1.3 | 14.4 |

**Appendix A**

**Version 4.0 Unique Identifying Number:**

**Translational Research for Spinal Cord Injury (SCI): Current and Future Perspectives**

**We are interested in establishing your views and thoughts about the current developments in science aimed at overcoming some of the primary and secondary complications spinal cord injury produces (e.g. mobility problems, pain, bladder, bowel and sexual functions).**

**In the following questions, please indicate your answer by putting a tick ✓ in the box like this: 🗹**

**SECTION 1: SOME QUESTIONS ABOUT YOU**

**1. How would you identify yourself religiously?**

**🞏** Christian (non catholic)**🞏** Catholic**🞏** Jewish**🞏** Islamic**🞏** Other Please State:

**🞏** No religion

**2. How would you describe your relationship status?**

**🞏** Single

**🞏** Married

**🞏** In a relationship

**🞏** Other **(please state)**

**3. How would you describe your living arrangements?**

**(please tick all that apply)**

**🞏** Living alone

**🞏** Living with spouse/ partner

**🞏** Living with parents

**🞏** Living with children

**🞏** Living with friends/ housemates

**🞏** Other **(please state)**

**4. Do you have any children or other dependants? (if YES, please describe how many and how old they are)**

**5.** **How do you spend your time?**

**(a)** How many hours per week do you spend working in a job for which you get paid?

**________**Hours.

Occupation:

**(b)** How many hours a week do you spend in school/ college working towards a qualification (please include hours in class and studying)?

**________**Hours.

**(c)** How many hours per week do you spend in active home-making, including parenting, housekeeping, and food preparation?

**________**Hours.

**(d)** How many hours a week do you spend in home maintenance activities such as gardening, house repairs or home improvement?

**________**Hours.

**(e)** How many hours a week do you spend in recreational activities, such as sports, exercise, going to the cinema?

**________**Hours.

**SECTION 2: SOME QUESTIONS ABOUT YOUR SPINAL CORD INJURY (SCI) AND HOW IT AFFECTS YOU**

**1.** **How many hours in a typical 24-hour day do you have someone with you to provide physical assistance or personal care activities (such as eating, bathing, dressing, toileting etc)?**

Hours of paid assistance.

Hours of unpaid assistance (e.g. family or friends).

**2. Are you up and about regularly?**

**(a)** In a typical 24 hour day how many hours are you out of bed? **________**Hours.

**(b)** In a typical week how many days do you get out of the house and go somewhere? **________**Days.

**(c)** In the last **YEAR**, how many nights have you spent away from your home (excluding time in hospital)?

**(please tick)**

**🞏** None

**🞏** 1-2

**🞏** 3-4

**🞏** 5 or more

**3. Are you currently taking any medication? YES/ NO** **(delete as appropriate)**

If **YES**, please describe (names and amount taken) in as much detail as you can

**4. Do you currently have any secondary complications resulting from your injury? (please indicate all that apply and how often they cause you discomfort by putting a tick  in the box like this: )**

|  | **Not at all** | **Sometimes** | **Fairly often** | **Almost always** |
| --- | --- | --- | --- | --- |
| Pressure sores |  |  |  |  |
| Spasticity |  |  |  |  |
| Contractures |  |  |  |  |
| Sleeplessness |  |  |  |  |
| Fatigue |  |  |  |  |
| Urinary tract infections |  |  |  |  |
| Respiratory conditions |  |  |  |  |
| Pain |  |  |  |  |
| Other **(please state)** |  |  |  |  |

**SECTION 3: SOME QUESTIONS ABOUT HOW YOU FEEL ABOUT YOUR LIFE AND YOUR SCI**

**The following section is designed to look at how you feel about your life in general. Below are a number of statements concerning how satisfied you are with different aspects of your life. For each of these statements please indicate how you feel by ticking the appropriate box.**

|  | **Very dissatisfying** | **Dissatisfying** | **Rather dissatisfying** | **Rather satisfying** | **Satisfying** | **Very satisfying** |
| --- | --- | --- | --- | --- | --- | --- |
| My life as a whole is |  |  |  |  |  |  |
| My vocational situation is |  |  |  |  |  |  |
| My financial situation is |  |  |  |  |  |  |
| My leisure situation is |  |  |  |  |  |  |
| My contact with friends and acquaintances is |  |  |  |  |  |  |
| My sexual life is |  |  |  |  |  |  |
| My ability to manage my self-care *(dressing, hygiene, transfers, etc)* is |  |  |  |  |  |  |
| My physical health is |  |  |  |  |  |  |
| My psychological health is |  |  |  |  |  |  |
| My family life is |  |  |  |  |  |  |
| My partner relationship is |  |  |  |  |  |  |

| I have no family |  |  |
| --- | --- | --- |
|  | |  |
| I have no steady partner relationship |  |  |

**The following questions ask how often you have certain feelings/ beliefs about your injury. Please read each statement carefully, indicating how often you feel like this by ticking the appropriate box.**

|  | **Not at all** | **Sometimes** | **Fairly often** | **Almost always** |
| --- | --- | --- | --- | --- |
| I believe this situation is manageable |  |  |  |  |
| I feel I have learnt the skills to cope with most of the  problems that have arisen from my injury |  |  |  |  |
| I believe I am able to continue to take part in activities  that I find enjoyable and rewarding |  |  |  |  |
| I feel supported by people around me |  |  |  |  |
| I am motivated to engage in what happens around me |  |  |  |  |

**SECTION 4: SOME QUESTIONS ABOUT HOW YOU FEEL ABOUT SCIENTIFIC RESEARCH, EXPERIMENTAL TREATMENTS AND CLINICAL TRIALS FOR SCI**

**Read the following statements and tick the box that most accurately describes how you feel.**

**Statement 1: I would like to be more involved in scientific research into SCI, including:**

|  | **Strongly agree** | **Agree** | **Mildly agree** | **Mildly disagree** | **Disagree** | **Strongly disagree** |
| --- | --- | --- | --- | --- | --- | --- |
| Receiving postal communications on scientific research into SCI |  |  |  |  |  |  |
| Attending scientific presentations |  |  |  |  |  |  |
| Participating in more questionnaires |  |  |  |  |  |  |
| Participating in patient involvement group meetings |  |  |  |  |  |  |
| Consenting for scientific researchers to access my patient records |  |  |  |  |  |  |
| Donating blood for research purposes |  |  |  |  |  |  |
| Donating saliva for research purposes |  |  |  |  |  |  |
| Donating fat for research purposes |  |  |  |  |  |  |
| Donating bone marrow for research purposes |  |  |  |  |  |  |
| Donating sperm samples for research purposes |  |  |  |  |  |  |
| Other **(please state)** |  |  |  |  |  |  |

**Would you like to be contacted for participation in future scientific research into SCI?**

**YES/ NO (delete as appropriate)**

**Statement 2: I would consider taking part in an experimental treatment or clinical trial that involved the use of:**

|  | **Strongly agree** | **Agree** | **Mildly agree** | **Mildly disagree** | **Disagree** | **Strongly disagree** |
| --- | --- | --- | --- | --- | --- | --- |
| Cells derived from animal tissues |  |  |  |  |  |  |
| Cells derived from  human embryos *(up to 8 weeks old)* |  |  |  |  |  |  |
| Cells derived from human foetuses *(up to 4 months old)* |  |  |  |  |  |  |
| Cells derived from human umbilical cords/ placentas |  |  |  |  |  |  |
| Cells derived from adult human donor tissues *(a stranger)* |  |  |  |  |  |  |
| Cells derived from adult human donor tissues *(a friend or relative)* |  |  |  |  |  |  |
| Cells derived from my own tissues |  |  |  |  |  |  |
| Cells that have been genetically modified |  |  |  |  |  |  |
| New experimental drugs |  |  |  |  |  |  |
| Electrical stimulation therapy *(e.g. external electrical impulses applied to your spine, arms and legs)* |  |  |  |  |  |  |
| Hypothermic therapy *(cooling of your spine)* |  |  |  |  |  |  |
| Physical therapy *(e.g. assisted movement of paralysed regions)* |  |  |  |  |  |  |
| Other **(please state)** |  |  |  |  |  |  |

**Statement 3: I would probably be prepared to take part in an experimental treatment or clinical trial which had NOT first been tested on:**

|  | **Strongly agree** | **Agree** | **Mildly agree** | **Mildly disagree** | **Disagree** | **Strongly disagree** |
| --- | --- | --- | --- | --- | --- | --- |
| Other people |  |  |  |  |  |  |
| Laboratory primates |  |  |  |  |  |  |
| Laboratory rodents |  |  |  |  |  |  |
| Any laboratory animals |  |  |  |  |  |  |

**Statement 4: I would probably be prepared to take part in an experimental treatment or clinical trial which involved:**

|  | **Strongly agree** | **Agree** | **Mildly agree** | **Mildly disagree** | **Disagree** | **Strongly disagree** |
| --- | --- | --- | --- | --- | --- | --- |
| Harvesting my blood |  |  |  |  |  |  |
| Harvesting my bone marrow |  |  |  |  |  |  |
| Harvesting my fat |  |  |  |  |  |  |
| Harvesting tissue from my olfactory bulb *(the front part of the brain responsible for your sense of smell).*  If you agree a small biopsy would be taken through nasal access under general anaesthetic. |  |  |  |  |  |  |
| Lumbar puncture *(spinal tap under local anaesthetic)* |  |  |  |  |  |  |
| Back surgery *(under general anaesthetic)* |  |  |  |  |  |  |

**Statement 5: In order to monitor whether an experimental treatment or clinical trial (before, during and after) was making a difference I would probably be prepared to take part in:**

|  | **Never** | **Once (after 12 months)** | **Monthly (for 12 months)** | **Weekly (for 12 months)** | **Whatever the trial might require** |
| --- | --- | --- | --- | --- | --- |
| Physical therapy |  |  |  |  |  |
| Neurological assessments |  |  |  |  |  |
| MRI imaging |  |  |  |  |  |
| Keeping a diary/ log book |  |  |  |  |  |

**Statement 6: I would probably take part in an experimental treatment or clinical trial if it was a 50:50 chance whether I received the trial drug/cell or a placebo.**

*(A placebo is, for example a tablet which looks the same but does not contain any active drug)*

**🞏 Strongly agree**

**🞏 Agree**

**🞏 Mildly agree**

**🞏 Mildly disagree**

**🞏 Disagree**

**🞏 Strongly disagree**

**Statement 7.** **I would like to be contacted for potential recruitment into a clinical trial at my UK spinal injury centre?**

**YES/ NO (delete as appropriate)**

**In the following questions, please indicate your answer by putting a tick ✓ in the box like this: 🗹**

**1. There are sometimes risks associated with experimental treatments and clinical trials being offered for SCI. If the following risk of a treatment was explained to you by a clinician before volunteering for an experimental treatment or clinical trial, how likely would you then be to take part?**

|  | **Very likely** | **Likely** | **Unlikely** | **Very unlikely** |
| --- | --- | --- | --- | --- |
| Tumour formation |  |  |  |  |
| Infection *(such as meningitis)* |  |  |  |  |
| Neurological worsening – this might include loss of motor function, bladder control or sexual function |  |  |  |  |
| Increased spasticity |  |  |  |  |
| Increased neuropathic pain |  |  |  |  |

**2. Have you ever considered going to a clinic for an experimental treatment or clinical trial not currently available on the NHS or in the UK?**

**🞏 Never**

**🞏 Once**

**🞏 A few times**

**🞏 Many times**

**3. If you have, where did you hear about these?**

|  | **Never** | **Once** | **A few times** | **Many times** |
| --- | --- | --- | --- | --- |
| Newspaper/ Magazine |  |  |  |  |
| Television News |  |  |  |  |
| Television Advertising |  |  |  |  |
| Internet News |  |  |  |  |
| Internet Advertising |  |  |  |  |
| Friends/ relatives |  |  |  |  |
| Other **(please state)** |  |  |  |  |

**4. Have you asked an NHS professional for their opinion on an experimental treatment or clinical trial available outside the NHS/ UK? If YES, how satisfied were you with the advice that you received?**

|  | **Very dissatisfied** | **Dissatisfied** | **Rather dissatisfied** | **Rather satisfied** | **Satisfied** | **Very satisfied** |
| --- | --- | --- | --- | --- | --- | --- |
| Your spinal injury consultant |  |  |  |  |  |  |
| Your GP |  |  |  |  |  |  |
| A nurse |  |  |  |  |  |  |
| A physiotherapist |  |  |  |  |  |  |
| An occupational therapist |  |  |  |  |  |  |
| A mental health professional |  |  |  |  |  |  |
| A SCI mentor |  |  |  |  |  |  |
| NHS Direct |  |  |  |  |  |  |
| Other **(please state)** |  |  |  |  |  |  |
